# Ozone Susceptibility of Tropical Tree Species

**DOI:** 10.1111/pce.70777

**Published:** 2026-08-02

**Authors:** Mst Nahid Farha, Flossie Brown, Lucas A. Cernusak, Stephen Sitch, Alexander W. Cheesman

**Affiliations:** ^1^ College of Science & Engineering and Centre for Tropical Environmental and Sustainability Science, James Cook University Cairns Queensland Australia; ^2^ Department of Chemistry Rajshahi University of Engineering &Technology Rajshahi Bangladesh; ^3^ Institute for Atmospheric and Climate Science, ETH Zurich Zurich Switzerland; ^4^ Faculty of Environment, Science and Economy University of Exeter Exeter UK

**Keywords:** leaf mass per area (LMA), O_3_‐dose response functions, ozone (O_3_), plant mass per unit leaf area (PMA), total antioxidant capacity (TAC), water‐use efficiency (iWUE)

## Abstract

Tropical forests account for approximately 60% of global terrestrial photosynthesis, yet their function into the future may be compromised by rising ground‐level ozone (O_3_) pollution. Although O_3_ is known to impair plant productivity, the physiological mechanisms underlying interspecific variation in O_3_ susceptibility—especially among tropical trees—remain poorly resolved. Here we exposed 11 tropical tree species to a gradient of O_3_ concentrations using open‐top chambers and quantified whole‐plant biomass responses alongside key plant functional traits. Species varied markedly in O_3_ susceptibility, with relative growth reductions best explained by differences in plant mass per unit leaf area (PMA). Contrary to expectations, antioxidant capacity and leaf mass per area were not predictive of O_3_ susceptibility of tropical tree species. These findings indicate that fundamental structural traits may mediate tropical tree responses to O_3_, and that species with higher PMA exhibit greater resilience—within the experimental framework employed here. Our results help inform trait‐based risk assessments and support the need for improved representation of O_3_ impacts in models of carbon cycling in tropical forests.

## Summary statement

We examined ozone (O_3_) susceptibility of 11 tropical tree species. Our findings show that structural traits mediate O_3_ susceptibility. These inform trait‐based risk assessments and highlight the need to better represent O_3_ impacts in tropical forest carbon cycling models.

**Abbreviations**
AOT_40_
: accumulated O_3_ concentrations above threshold of 40 ppb
CO: carbon monoxide
DO_3_SE: deposition of O_3_ for stomatal exchange
FRAP: ferric reducing antioxidant power
*g*_max_
: maximum stomatal conductance
*g*_min_
: minimum stomatal conductance
*g*_s_
: stomatal conductance
iWUE: intrinsic water‐use efficiency
LAR: leaf area ratio
LMA: leaf mass per area
LMA_Lamina_
: lamina leaf mass per area
LMR: leaf mass ratio
NOx: nitrogen oxides
O_3_
: ozone
PAR: photosynthetically active radiation
PMA: plant mass per area
POD_1_
: phytotoxic O_3_ dose‐the accumulated stomatal O_3_ flux above a threshold of 1nmol m^−2^ s^−1^
RH: relative humidity
RuBisCO: ribulose 1,5‐bisphosphate carboxylase/oxygenase
R:S: root‐to‐shoot ratio
T: air temperature
TAC: total antioxidant capacity
TPC: total phenolic content
TPTZ: 2,4,6‐tripyridyl‐s‐triazine
VOC: volatile organic compound
VPD: vapour pressure deficit
W126: sigmoidal weighted cumulative O_3_ exposure index
δ^13^C: carbon stable isotope ratio
[O_3_]: ozone concentration
[O_3_]_day_
: daytime mean O_3_ concentration

## 1 Introduction

Ozone (O_3_) in the lower atmosphere (troposphere) is a secondary air pollutant that has detrimental impacts on plant growth across the globe, reducing the productivity of natural systems and crops worldwide (Ainsworth et al. 2012; Emberson 2020). Ozone is generated naturally in the atmosphere by a series of photochemical reactions between precursors often elevated by anthropogenic activities (Jacob 2000; Dewan and Lakhani 2022). Across the tropics, rapid urbanisation, industrialisation, and rising energy consumption have driven increases in the emission of these precursors (e.g., nitrogen oxides (NO_x_), carbon monoxide (CO) and volatile organic compounds [VOCs]) (Zhang et al. 2021). Given their long range transport, this has resulted in a pervasive increases in ground‐level O_3_ across even intact forested regions (Gaudel et al. 2024). Modelling studies predict that O_3_ concentrations across the tropics will continue to rise, driven by likely socioeconomic pathways and the additional climate‐change penalty (Griffiths et al. 2021; Brown et al. 2022; Dewan and Lakhani 2022). This elevated, and increasing, concentration of O_3_ will continue to have negative impacts on tropical plant productivity and growth, as O_3_ causes both acute and chronic oxidative stress (Ainsworth et al. 2012; Emberson 2020).

Tropical forests are a major component of the global carbon budget, however their future role as a sink in the carbon cycle is uncertain due to the pressures of changing land‐use and global environmental change (Uribe et al. 2023)—including the often under‐appreciated impact of ground‐level O_3_ (Sitch et al. 2007; Cheesman et al. 2024). An Earth‐system models calibrated to an average response of tropical trees to O_3_ suggests that anthropogenic increases in O_3_ have caused a substantial decline in productivity of extant forests, resulting in the cumulative loss in carbon drawdown of 0.29 PgC year^−1^ since 2000—equivalent to aproximately 17% of the contemporary annual tropical land sink in the 21st century (Cheesman et al. 2024). Although the negative effects of O_3_ on tropical plant productivity may be substantial, likely resulting in an indirect impact on global climate change (Sitch et al. 2007), the exceptional species richness of tropical forests means that to accurately predict ecosystem‐scale losses we need to understand how these impacts will emerge from the physiological variation observed among tropical tree species. This understanding will subsequently enable the use of trait‐based approaches to account for tropical biodiversity within global models (Ma et al. 2023).

Plants exposed to O_3_ show responses across a range of processes and scales, consistent with species‐specific responses to elevated oxidative stress (Bergmann et al. 2017; Grulke and Heath 2020). However, typical O_3_ impacts include decreased photosynthesis (Wittig et al. 2007), stomatal sluggishness (Hoshika et al. 2015), symptoms of visible injury (Feng et al. 2014; Moura et al. 2018) and premature leaf senescence (Nunn et al. 2005), which in combination result in reduced carbon gain and plant growth (Wittig et al. 2009). Consequently, net biomass accumulation—reflecting whole‐plant O_3_ susceptibility—has been widely used in species‐level risk assessments across both O_3_ concentration‐ and flux‐based metrics (Emberson et al. 2009; Büker et al. 2015; Lee et al. 2022). These approaches have highlighted significant inter‐ and intra‐specific variation in O_3_ susceptibility of extratropical species (Büker et al. 2015; Shang et al. 2020; Lee et al. 2022).

Variation in O_3_ susceptibility arises due to variation in plant functional traits (Bussotti 2008; Li et al. 2016), the nature of which will differ depending on whether susceptibility has been defined as a response to a concentration or flux‐based exposure metric. If considering susceptibility using an O_3_ concentration based metrics (e.g., AOT_40_—accumulated time above a threshold of 40 ppb, or the sigmoidally‐weighted W126 exposure index (Lefohn and Runeckles 1987)) then all else being equal, plants with high stomatal conductance (*g*_s_) will likely be found to be more susceptible to O_3_, as a higher *g*_s_ means a higher O_3_ flux to the intercellular air space (Reich 1987; Paoletti 2006). However, when moving to flux‐based metrics which account for intrinsic and dynamic differences in *g*_s_ (Büker et al. 2015; Pleijel et al. 2022), then O_3_ susceptibility is believed to be linked to internal morphological and biochemical traits. All plants possess antioxidant defence systems that can mitigate O_3_ damage by scavenging the reactive oxygen species produced (Castagna and Ranieri 2009; Dusart et al. 2019). Species with higher antioxidant capacity, greater localisation in the apoplast, or with more efficient cycling of denatured antioxidants may therefore exhibit higher O_3_ tolerance due to their improved detoxification capacity (Feng et al. 2010; Inada et al. 2012; Yendrek et al. 2015). Similarly, high leaf mass per area (LMA) has been consistently associated with reduced O_3_ susceptibility, potentially because thicker leaves dilute the O_3_ burden per unit mass (Calatayud et al. 2011; Zhang et al. 2012; Li et al. 2016; Feng et al. 2018). Indeed a meta‐analysis of subtropical, temperate and Mediterranean tree species has shown that interspecific variation in O_3_ susceptibility among woody species can, to a large extent, be explained by variation in LMA (Feng et al. 2018). However, whether these relationships extend to tropical trees remains unknown.

Furthermore, the influence, and interaction, of other key leaf and plant‐level functional traits, such as intrinsic water‐use efficiency (iWUE) and leaf nitrogen concentration (N‐leaf), on O_3_ responses are poorly understood. Higher iWUE reflects lower water loss, and therefore lower O_3_ uptake per unit carbon gained, potentially lowering oxidative stress risk. Conversely, high N‐leaf is directly linked to photosynthetic capacity, through its role in RuBiSCO (Wright et al. 2004). Species with greater N investment may support higher CO_2_ assimilation rates but simultaneously increase the flux of O_3_ into leaves, potentially exacerbating oxidative damage. Together, these traits represent a functional axis balancing carbon gain and O_3_ exposure, and may help explain interspecific variation in observed O_3_ susceptibility.

In this study, 11 tropical tree species were grown in open top chambers under a range of biologically relevant O_3_ concentrations [O_3_]. The main objectives were to:

i. Evaluate changes in functional and morphological traits of tropical trees in response to O_3_ exposure.
ii. Quantify interspecific variation in O_3_ susceptibility, using whole‐plant biomass response as an integrated measure.
iii. Identify the key traits or trait combinations that best explain this variation, to reveal potential functional strategies underlying O_3_ tolerance.

This work builds directly upon Cheesman et al. (2024), which quantified an average O_3_ susceptibility across 10 of the 11 tropical tree species that we have examined in this paper. Here, by linking interspecific trait variation to those observed responses to O_3_, we seek to provide a mechanistic basis for predicting how diverse tropical forests may respond to rising tropospheric O_3_.

## 2 Materials and Methods

### 2.1 Experimental Site and Ozone Exposure

The O_3_ exposure experiment was conducted at the joint University of Exeter (UoE) and James Cook University (JCU) TropOz research facility (www.tropoz.org) located at the James Cook University's Environmental Research Complex (www.jcu.edu.au/environmental-research-complex) on the Nguma‐bada campus in far‐north Queensland, Australia. This experimental facility consists of nine independently controlled and monitored open top chambers which allows the examination of tropical tree species grown under different O_3_ concentrations. The chambers (internal volume 22.2 m^3^) are ventilated with charcoal filtered air augmented with O_3_ generated on site and supplied to each chamber between 8:00 and 17:00 to obtain a range of nine different O_3_ exposures, see Cheesman et al. (2024) for details. In addition to monitoring chamber O_3_ concentrations in each chamber using a UV spectrometer (Model 205, 2B Tech, Broomfield, CO, USA), meteorological conditions including air temperature (T), relative air humidity (RH), shortwave radiation and photosynthetically active radiation (PAR) were recorded using a single meteorological monitoring station (Campbell Scientific, Logan UT, USA) established in the central open top chamber.

### 2.2 Plant Material

Eleven tropical angiosperm tree species drawn from 10 families (Table 1) were selected. The species differ in key leaf morphological traits (e.g., individual leaf area, LMA) and represent a range of forest successional stages, allowing comparative evaluation of O_3_ susceptibility across a range of tropical species. All seedlings were sourced locally and planted when approximately 20 cm tall into either 20 or 60 L pots filled with locally sourced ‘garden mix’ topsoil mixed 3:1 with Quincan (a local form of scoria) to improve drainage. Potted saplings were acclimatised to full sun for approximately a month before three or four replicate plants of each species were transferred into each open top chamber. Individual species were introduced to the open top chambers in a rolling experimental programme and plants grown for between 61 and 198 (average = 152) days (Table 1). Plants were irrigated daily using individual drip irrigation and fertilised as required using a controlled release fertiliser (Osmocote Native Formula, ScottsMicacle‐Gro, Marysville, OH, USA).

**Table 1:** Eleven tropical tree species grown as part of O_3_ susceptibility experiments in open top chambers at the TropOz experimental facility on the Naguma‐bada campus of James Cook University. For more details see the Australian Tropical Rainforest Key (Zich et al. 2020).

Species	Family	Ecological niche	O_3_ fumigation	
			Start	End
*Brachichyton acerifolius*	Malvaceae	Endemic to Australia, brevi‐deciduous as a mature tree found in drier, more seasonal well developed rain forests	10/09/2021	10/11/2021
*Carallia brachiata*	Rhizophoraceae	Grows in well‐developed rain forest, dry rain forest, gallery forest and rain forest around swamps	25/05/2022	17/11/2022
*Calophyllum inophyllum*	Clusiaceae	Pan‐tropical. Often found growing near beaches	08/04/2021	24/09/2021
*Carica papaya*	Caricaceae	Pan‐tropical fast‐growing pioneer cultivated crop species	18/05/2022	2/12/2022
*Chionanthus ramiflorus*	Oleaceae	Late successional tree found in rain forest, seasonal rain forest, monsoon forest and beach forests.	18/05/2022	18/11/2022
*Darlingia darlingiana*	Proteaceae	Endemic to North East Queensland, but locally common. Grows in well‐developed rain forest on a variety of sites. And is favoured by disturbance	28/03/2022	05/10/2022
*Flindersia pimenteliana*	Rutaceae	Late successional tree found in well developed rain forest on a variety of sites.	30/07/2022	30/11/2022
*Homalanthus novo‐guineensis*	Euphorbiaceae	Early successional pioneer species favoured by disturbance	14/07/2020	10/12/2020
*Inga edulis*	Fabaceae	Pan‐tropical nitrogen‐fixing cultivated tree crop ‐ often found in agroforestry systems.	28/4/2021	19/08/2021
*Syzygium gustavioides*	Myrtaceae	Endemic to North East Queensland, Late successional tree found in well developed rain forests.	01/05/2022	05/10/2022
*Theobroma cacao*	Malvaceae	Pan‐tropical cultivated tree crop, considered late successional	13/07/2020	13/01/2021

### 2.3 Sampling and Trait Characterisation

Stomatal function: In the weeks prior to harvest, measurements of *g*_s_ were made on the youngest fully expanded mature leaf of all trees across the range in O_3_ exposure using an SC‐1 Leaf Porometer (Decagon Devices, Pullman, WA, USA). Point measurements of *g*_s_ on both abaxial and adaxial leaf surfaces were collected over a range of time and weather conditions and coupled to the closest meteorological data as recorded by the experimental system. These values were used to parameterise the DO_3_SE model (Deposition of O_3_ for Stomatal Exchange, version 3.1, https://www.sei.org/tools/do3se-deposition-ozone-stomatal-exchange/, Supporting Information: Table S1), using the method described by (Hayes et al. 2020), allowing for the calculation of Phytotoxic O_3_ Dose above a threshold of 1 nmol m^−2^ s^−1^ (POD_1_) as per CLRTAP et al. (2017).

Leaf functional traits: Between 6 and 12 fully expanded mature leaves (leaf number dependent upon leaf size) were collected from every plant at the end of the O_3_ exposure period. Leaves were scanned to determine leaf area and shape, weighed to determine fresh mass, and half were dried (70°C, 72 h) for determination of whole leaf‐LMA. In the remaining fresh leaves, lamina tissue (without midrib or major veins) was excised using a scalpel and rescanned. Lamina tissue was then wrapped in tin foil and snap‐frozen in liquid N_2_ to await lyophilization. After drying, samples were weighed to determine lamina LMA (LMA_Lamina_) and used to determine biochemical traits (see below).

Biomass: At the end of the experiment, plants were harvested for total biomass and separated into leaves, stems, and roots to calculate biomass partitioning. Dry biomass was determined after oven‐drying at 70°C until constant weight was achieved. Oven dried leaf biomass and total plant biomass were used to calculate the leaf mass ratio (LMR) of each plant [LMR = leaf biomass/total plant biomass, g g^−1^]. Plant‐level leaf area ratio (LAR) was calculated using the LMR and leaf LMA measurements [LAR = LMR/LMA, m^2^ g^−1^], alongside its inverse plant mass per unit leaf area (PMA in unit g m^−2^).

Physiological and biochemical characterisation: Freeze‐dried leaf lamina samples were used to determine physiological and biochemical traits such as iWUE calculated from δ^13^C, leaf nitrogen (N‐leaf), total antioxidant capacity (TAC) and total phenolic content (TPC). To do this, dried leaf samples were ground into fine powder (Rocklabs Bench Top Ring Mill, Scott, Dunedin New Zealand) and stored in airtight vials.

The δ^13^C and N‐leaf were determined by using a Costech Elemental Analyser fitted with a zero‐blank auto‐sampler coupled via a ConFloIV to a ThermoFinnigan DeltaVPLUS using Continuous‐Flow Isotope Ratio Mass Spectrometry (EA‐IRMS) at James Cook University's Advanced Analytical Centre. The δ^13^C results are reported as per mil (‰) deviations from the VPDB reference. Precisions (S.D.) on internal standards were better than 0.1‰ for δ^13^C. The N‐leaf results were determined as weight percent (%). We calculated iWUE from δ^13^C values using the equation of Farquhar et al. (1989).

Total antioxidant capacity (TAC) was measured by the ferric reducing antioxidant power (FRAP) assay (Benzie and Strain 1996) with some modifications. Leaf samples (~ 30 mg) were extracted in cold 50% acetone (Ritmejerytė et al. 2019). Fresh FRAP reagent was prepared by mixing 300 mM acetate buffer (pH 3.6), 10 mM TPTZ (2,4,6‐tripyridyl‐s‐triazine) solution (in 40 mM HCl) and 20 mM FeCl_3_.6H_2_O solution, which was warm at 37°C in a water bath until used. The volume ratio of the acetate buffer, TPTZ and FeCl_3_.6H_2_O solutions was 10:1:1 respectively. Ascorbic acid was used to make a standard calibration curve in the range of 0–250 µg mL^−1^. Leaf extracts (10 µL) were placed in a 96‐well plate to react with the FRAP solution (190 µL) for 30 min in the dark. The absorbance of the reaction mixture was then measured at 593 nm with a microplate reader (FLUOstar OPTIMA, BMG LABTECH Pty. Ltd). Results were expressed as ascorbic acid equivalents (mg AAE g^−1^ dry weight).

Total phenolic content (TPC) was determined from the same acetone leaf extract using the Folin–Ciocalteu method (Singleton and Rossi 1965; Cork and Krockenberger 1991), with minor modifications as described by Ritmejerytė et al. (2019). Initially, 20 μL of leaf extract was mixed with 380 μL of distilled water and 25 μL of Folin–Ciocalteu reagent. After 3 min, 75 μL of 20% Na_2_CO_3_ (w/v) was added, and the reaction mixture was incubated at room temperature for 20 min. Absorbance was then measured at 765 nm using a microplate reader (FLUOstar OPTIMA, BMG LABTECH Pty. Ltd, VIC, Australia). TPC was quantified against a gallic acid standard curve and expressed as gallic acid equivalents (mg GAE g^−1^ dry weight).

### 2.4 Ozone Dose–Response Functions

To determine species susceptibility to O_3_ we used a gradient analysis (Kreyling et al. 2018) using both a concentration and flux based metrics of O_3_ exposure. Daytime mean O_3_ concentration, [O_3_]_day_ (from 08:00 to 17:00) in each chamber was computed for all species/chambers while we also calculated POD_1_ using species‐specific parametrization of the DO_3_SE model v 3.1. Specifically, we used estimates of maximum *g*_s_ (*g*_max_), effective leaf width (L_d_), and empirical *g*_s_ model fitting parameters (i.e., f_min_, f_PAR_, f_VPD_, f_temp_, Supporting information: Table S1) used in conjunction with experiment specific meteorological data (Supporting information: Table S2) to calculate chamber levels of POD_1_ (Supporting information: Table S3).

### 2.5 Statistical Analyses

All statistical analyses were conducted using the software R version 4.5.1 (R Core Team 2025). To evaluate the effects of O_3_ exposure on leaf physiological and structural traits, we fitted linear mixed‐effects models using the *lmer* function from the lme4 package in R (Bates et al. 2015). The leaf traits analysed included TAC, TPC, LMA_Lamina_, and iWUE while structural traits included LMR and R:S. For each trait, two separate models were constructed with either [O_3_]_day_ or POD_1_ as fixed effects. Species was included as a random intercept to account for species‐level variation. This approach allowed us to estimate generalisable effects of O_3_ exposure on tropical trees. Type III Wald chi‐square tests were used to evaluate the statistical significance of fixed effects using the *ANOVA()* function from the car package (Fox and Weisberg 2019). Model assumptions were assessed by examining residual plots for normality and homoscedasticity, and model summaries were inspected for convergence and singular fit issues.

To assess the impact of O_3_ exposure on overall plant growth, we modelled relative biomass as a function of both [O_3_]_day_ and POD_1_. Biomass was first averaged at the chamber level within species to avoid pseudoreplication, and relative biomass (%) was calculated by scaling chamber‐mean biomass values to the fitted intercept (predicted biomass at zero O_3_ exposure). Linear models were fitted with O_3_ exposure and species as fixed effects, and model performance was assessed via adjusted *R* ^2^, residual standard error, and Type II ANOVA. To quantify species‐specific dose–response relationships and their associated uncertainty, we used the *emtrends()* function from the emmeans package (Lenth 2025) to extract estimated marginal slopes (± SE) of relative biomass against O_3_ exposure for each species. These slopes were interpreted as comparative indices of species susceptibility to increasing O_3_ exposure.

To explore coordination among plant functional traits and their association with O_3_ susceptibility, we first averaged traits across all individuals of a species, and then examined relationships between traits using pairwise Pearson correlation coefficients among six leaf‐ and plant‐level traits: LMR, PMA, LMA_Lamina_, TPC, iWUE, and N‐leaf content. Correlation matrices were visualised using the *ggpairs()* function from the GGally package in R (Schloerke et al. 2025). Significant associations (*p* < 0.05) were used to infer key trait trade‐offs and covariation patterns. Strongly collinear trait pairs (LMR to PMA and LMA_Lamina_ to N‐leaf) were identified and one variable from each pair excluded to avoid multicollinearity, leaving PMA, LMA_Lamina_, iWUE, and TPC as candidate predictors in subsequent regression models. To identify functional traits best explaining variation in species‐level O_3_ susceptibility (expressed as dose–response functions for both [O_3_]_day_ and POD_1_), all candidate predictors were standardised to z‐scores (mean = 0, SD = 1) prior to analysis, ensuring that coefficients were directly comparable. Exhaustive model selection was then performed using the bestglm package (McLeod et al. 2020) with AIC as the selection criterion. As the best‐supported models retained a single predictor, assessment of variance inflation factors was not required. Model fit was assessed using adjusted *R* ^2^, F‐statistics, and residual diagnostics to confirm that model assumptions were met.

## 3 Results

In a series of experiments, we examined the O_3_ susceptibility of 11 tropical tree species, drawn from 10 families. These species represented a broad range of leaf morphological characteristics and successional stages, with an average leaf (or leaflet) area from 17 cm^2^ in *Flindersia pimentelliana* (Rutaceae) to 795 cm^2^ in *Homolanthus novo‐guineensis* (Euphorbiaceae) (Table 2), and with an LMA_Lamina_ of 40 ± 2.8 g m^−2^ in *C. papaya* (Caricaceae) to 143 ± 5.3 g m^−2^ in *Calophyllum inophyllum* (Clusiaceae) (Table 2). Within each species, plants were exposed to nine different concentration levels of O_3_ (Supporting Information: Table S3). Due to temporal changes in ambient O_3_, and variation in the performance of the O_3_ control system between experimental runs, levels of exposure were not the same across experiments, however on average [O_3_]_day_ ranged from 19.4 to 112 ppb, and AOT_40_ ranged from 0 to 100 ppm‐h (Supporting Information: Table S3).

**Table 2:** Leaf‐level functional traits in 11 tropical tree species used in this study including average size of sampled leaves (Leaf size), leaf mass per area of the lamina (LMA_Lamina_), total phenolic content (TPC), total antioxidant capacity (TAC), intrinsic water‐use efficiency (iWUE), leaf nitrogen content (N‐leaf), leaf mass ratio (LMR) and plant mass per unit area (PMA). Data mean ± SD (*n* = 18–36 individual plants grown across a range in O_3_ exposure).

Species	Leaf size (cm^2^)	LMA_Lamina_ (g m^−2^)	TPC (GAE g^−1^)	TAC (AAE g^−1^)	iWUE (μmol mol^−1^)	N‐leaf (%)	LMR (g g^−1^)	PMA (g m^−2^)
*Brachichyton acerifolius*	276 ± 46.6	71.6 ± 4.17	33.3 ± 8.23	40.9 ± 12.6	88.2 ± 8.72	2.09 ± 0.30	0.451 ± 0.042	169 ± 16
*Carallia brachiata*	41.2 ± 4.98	71.3 ± 3.53	36.9 ± 7.37	39.7 ± 10.0	85.5 ± 10.4	2.17 ± 0.40	0.348 ± 0.047	214 ± 30
*Calophyllum inophyllum*	111 ± 17.3	143 ± 9.80	48.4 ± 7.26	37.7 ± 5.56	97.1 ± 19.5	1.16 ± 0.11	0.516 ± 0.043	302 ± 38
*Carica papaya*	202 ± 39.1	40 ± 2.84	27.2 ± 3.66	19.0 ± 4.21	72.4 ± 4.09	3.28 ± 0.33	0.088 ± 0.021	492 ± 186
*Chionanthus ramiflorus*	89.1 ± 25.4	117 ± 19.2	53.0 ± 9.34	50.6 ± 10.7	84.2 ± 8.85	1.50 ± 0.40	0.470 ± 0.047	271 ± 46
*Darlingia darlingiana*	233 ± 61.1	85.1 ± 15.0	138 ± 16.0	161 ± 22.4	82.0 ± 10.2	1.64 ± 0.57	0.484 ± 0.045	226 ± 39
*Flindersia pimenteliana*	16.5 ± 4.4	60 ± 8.86	64.7 ± 13.6	79.1 ± 16.8	81.3 ± 16.3	2.56 ± 0.29	0.522 ± 0.029	121 ± 15
*Homalanthus novo‐guineensis*	795 ± 94.1	86.6 ± 7.31	131 ± 11.3	192 ± 14.8	71.9 ± 6.97	2.46 ± 0.21	0.318 ± 0.021	310 ± 41
*Inga edulis*	105 ± 29.1	58.2 ± 5.19	68.8 ± 8.39	87.6 ± 12.7	44.8 ± 9.78	2.71 ± 0.27	0.535 ± 0.079	119 ± 32
*Syzygium gustavioides*	45.5 ± 9.88	113 ± 10.6	108 ± 19.6	157 ± 29.9	106 ± 6.11	2.05 ± 0.29	0.495 ± 0.048	246 ± 40
*Theobroma cacao*	243 ± 45.7	65.5 ± 9.18	58.1 ± 8.78	49.8 ± 6.49	76.4 ± 9.12	1.90 ± 0.26	0.438 ± 0.052	188 ± 43

### 3.1 Changing Leaf Traits With O_3_ Exposure

Total antioxidant capacity (TAC) and total phenolic content (TPC) were strongly and positively correlated across all sampled leaves (Figure 1), consistent with the well‐established role of phenolics as major contributors to antioxidant defence in plant tissues. Mixed‐effects modelling confirmed this relationship, with TAC explaining a substantial proportion of the variation in TPC (*β* = 0.619 ± 0.021 SE; *χ* ^2^ = 865, *df* = 1, *p* < 0.001). The relationship held across all experimental conditions, with relatively modest variance attributable to species identity (*σ* ^2^_species_ = 79.3) compared to residual variation (*σ* ^2^ = 36.0). Given this strong correspondence, we focused subsequent analyses on TPC as a representative stable indicator of antioxidant capacity in relation to O_3_ exposure.

**Figure 1:**
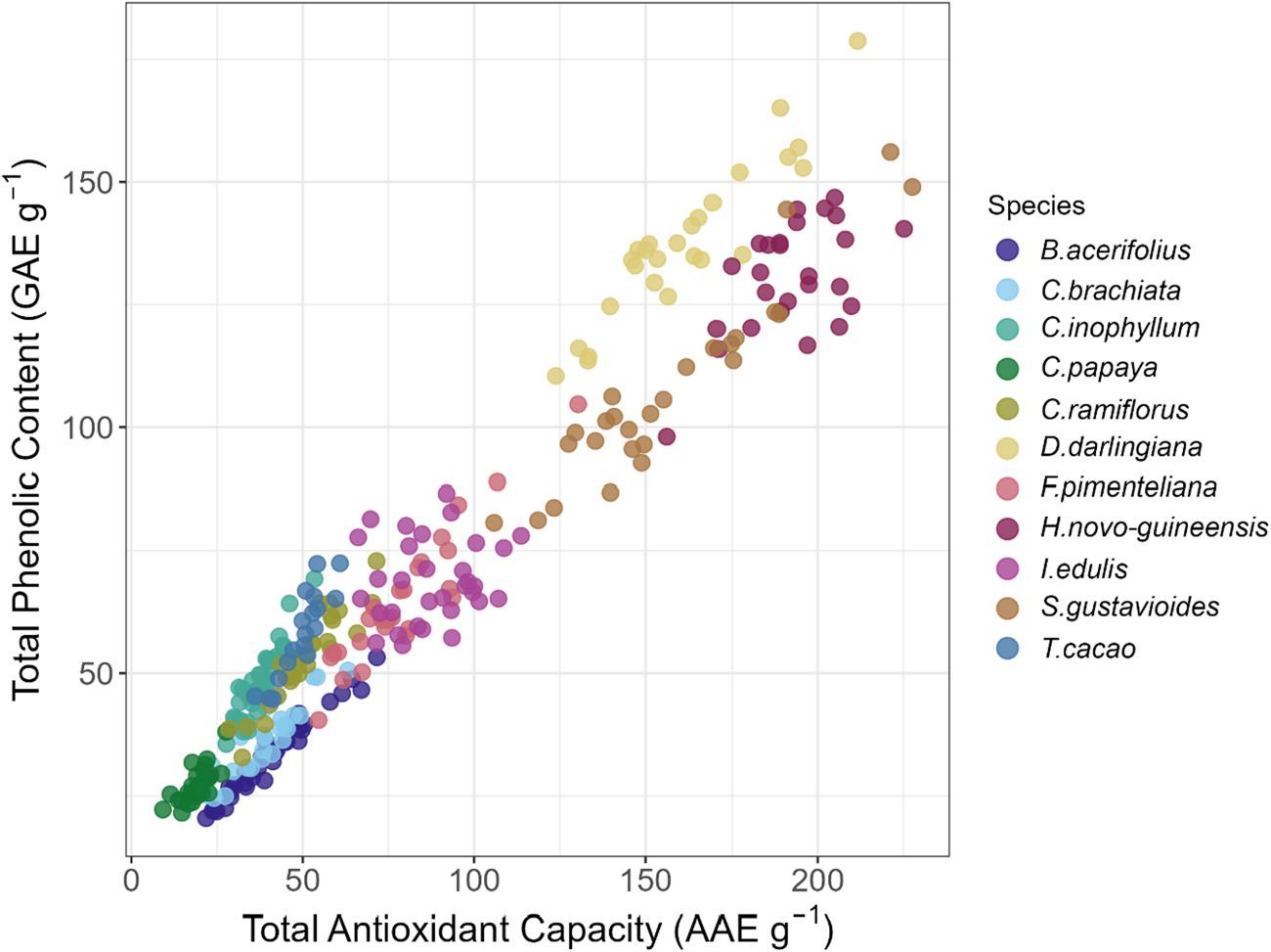
Leaf level total antioxidant capacity and phenolic content in 11 tropical tree species grown under a range of O_3_ exposures.

Total phenolic content (TPC) varied significantly among species tested (Table 2), but showed no consistent response to cumulative O_3_ exposure as expressed as either [O_3_]_day_ or POD_1_. Although the model including [O_3_]_day_ estimated an effect on TPC that was negative (*β* = –0.032 ± 0.02 SE), the relationship was not statistically significant (*χ* ^2^ = 2.6, *df* = 1, *p* = 0.11). Similarly, the model incorporating POD_1_ as a predictor showed no significant association with TPC (*χ* ^2^ = 1.9, *df* = 1, *p* = 0.17). The slope of the relationship was again negative (*β* = –0.044 ± 0.032 SE), but non‐significant, and overall model performance was similar to that of the [O_3_]_day_ model based on the REML criterion. Across both models, a large proportion of variance in TPC was attributable to differences among species, with the random effect for species explaining substantially more variance (*σ* ^2^_species_ = 1467 and 1463) than the residual error (*σ* ^2^ = 121). Scaled residuals were symmetrically distributed in both models, indicating no major violations of model assumptions.

Leaf mass per area of the lamina tissue (LMA_Lamina_) varied among species but showed no significant response to cumulative O_3_ exposure, whether quantified as [O_3_]_day_ or POD_1_. In the [O_3_]_day_ model, the estimated relationship with LMA_Lamina_ was negative (*β* = –0.015 ± 0.018 SE), but not statistically significant (*χ* ^2^ = 0.67, *df* = 1, *p* = 0.62). Similarly, the model including POD_1_ yielded a non‐significant association (*β* = –0.028 ± 0.029 SE; *χ* ^2^ = 0.98, *df* = 1, *p* = 0.32). In both models, variation in LMA was predominantly attributable to differences among species, with the random intercept for species accounting for substantially more variance (*σ* ^2^_species_ ≈ 920 and 916) than the residual error (*σ* ^2^ ≈ 96). Scaled residuals showed no substantial deviation from normality, indicating that model assumptions were satisfied.

Intrinsic water‐use efficiency (iWUE) showed no statistically significant response to O_3_ exposure. For [O_3_]_day_, the model estimated a positive but non‐significant effect (*β* = 0.017 ± 0.02 SE; *χ* ^2^ = 0.68, *df* = 1, *p* = 0.41). Similarly, the POD_1_ model produced a positive but non‐significant slope (*β* = 0.051 ± 0.032 SE; *χ* ^2^ = 2.50, *df* = 1, *p* = 0.11). Random intercept variance was *σ* ^2^_species_ = 242 (POD_1_ model: 250), with residual variance *σ* ^2^ ≈ 124 in both cases. Residuals showed no major departures from normality or homoscedasticity.

### 3.2 Response of Biomass Allocation to O_3_ Exposure

Exposure to O_3_ had significant effects on biomass allocation in tropical tree saplings. Leaf mass ratio (LMR) increased significantly with O_3_ exposure (Supporting information: Figure S1a,b). Using [O_3_]_day_, the model estimated a positive effect on LMR (*β* = 5.96 × 10^−4^ ± 8.22 × 10^−5^ SE; *χ* ^2^ = 52.61, *df* = 1, *p* < 0.001). A similar result was obtained using POD_1_, which showed a significant positive association (*β* = 8.92 × 10^−4^ ± 1.30 × 10^−4^ SE; *χ* ^2^ = 47.21, *df* = 1, *p* < 0.001). Random intercept variance for species was *σ* ^2^_species_ = 0.0168 (POD_1_ model: 0.0197), while residual variance was *σ* ^2^ ≈ 0.0018 in both models.

The root‐to‐shoot ratio (R:S) declined significantly across species with increasing O_3_ exposure (Supporting information: Figure S1c,d). For [O_3_]_day_ the model estimated a negative effect (*β* = −7.46 × 10^−4^ ± 1.62 × 10^−4^ SE; *χ* ^2^ = 21.20, *df* = 1, *p* < 0.001) while the POD_1_ model indicated a similar significant decrease (*β* = −1.13 × 10^−3^ ± 2.54 × 10^−4^ SE; *χ* ^2^ = 19.81, *df* = 1, *p* < 0.001). Random intercept variance for species was *σ* ^2^_species_ = 0.024 (POD_1_ model: 0.029), with residual variance *σ* ^2^ ≈ 0.007. Scaled residuals were broadly symmetric, with no evidence of heteroscedasticity.

### 3.3 Tropical Tree Susceptibility to O_3_ Exposure

Our statistical models provided substantial explanatory power (Adj‐*R* ^2^ = 0.65 and 0.63 for [O_3_]_day_ and POD_1_, respectively) with significant interactions (*p* < 0.001) between O_3_ exposure metric and species evident in both models, indicating that species differed in their susceptibility to O_3_ (Table 3). When these species‐level dose response functions and their confidence limits were examined, two species (*Carica papaya* and *Chionanthus ramiflorus*) were found to be O_3_ tolerant with no significant change in final biomass (Table 3). However, across the 11 tropical trees examined the average species‐specific dose response functions were −0.21% ppb^−1^ (IQR − 0.37 to −0.16% ppb^−1^) and −0.52% POD_1_^−1^ (IQR − 0.79 to −0.30% POD1^−1^) respectively.

**Table 3:** Ozone (O_3_) dose response functions of 11 tropical trees grown in open top chambers under a range of [O_3_] (Figure 2). Data fitted to two different O_3_ metrics daytime mean O_3_, [O_3_]_day_ and flux‐based metric POD_1_ (Phytotoxic O_3_ Dose ‐ the accumulated stomatal O_3_ flux above a threshold of 1 nmol O_3_ m^−2^ projected leaf area (PLA) s^−1^).

Species	Daytime [O_3_] (% ppb^−1^)	POD_1_ (% POD_1_^−1^)
*Brachichyton acerifolius*	−0.19 ± 0.10	−1.07 ± 0.55
*Carallia brachiata*	−0.24 ± 0.11	−0.37 ± 0.16
*Calophyllum inophyllum*	−0.21 ± 0.09	−0.32 ± 0.14
*Carica papaya*	0.05 ± 0.11	0.04 ± 0.09
*Chionanthus ramiflorus*	−0.07 ± 0.11	−0.14 ± 0.21
*Darlingia darlingiana*	−0.29 ± 0.12	−0.52 ± 0.21
*Flindersia pimentelia*	−0.16 ± 0.11	−0.62 ± 0.42
*Homalanthus novo‐guineensis*	−0.17 ± 0.15	−0.28 ± 0.25
*Inga edulis*	−0.50 ± 0.10	−0.80 ± 0.16
*Syzygium gustavioides*	−0.53 ± 0.12	−0.95 ± 0.21
*Theobroma cacao*	−0.46 ± 0.14	−0.79 ± 0.24

### 3.4 Plant Functional Traits Related to O_3_ Susceptibility

Pearson correlation analysis showed that LMR was negatively correlated with N‐leaf (−0.61, *p* < 0.05) and strongly negatively correlated with PMA (*r *= –0.80, *p* < 0.01). Leaf lamina (LMA_Lamina_) was strongly negatively correlated with both N‐leaf content (*r* = –0.83, *p* < 0.001) and positively with iWUE (*r* = 0.63, *p* < 0.05) (Supporting Information: Figure S2). To reduce redundancy and improve model interpretability, LMR and N‐leaf were excluded from the regression analysis, with PMA and LMA_Lamina_ retained as ecologically distinct and representative variables.

Exhaustive model selection using *bestglm()* identified PMA as the sole predictor in the best‐supported model for both O_3_ exposure metrics, consistent across all candidate model combinations. When O_3_ susceptibility was expressed as the [O_3_]_day_ dose–response function, PMA showed a positive association (standardised *β* = 0.109 ± 0.048 SE; *F*_1,9_ = 5.11, adjusted *R* ^2^ = 0.29), though this relationship was marginal (*p* = 0.050). When O_3_ susceptibility was expressed as the POD_1_ dose response function, PMA was again the strongest and sole retained predictor, with a notably stronger and significant model fit (standardised *β* = 0.263 ± 0.077 SE; *F*_1,9_ = 11.56, adjusted *R* ^2^ = 0.51, *p* < 0.01). In both models, the positive coefficient indicates that species with greater PMA tended to be less susceptible to O_3_ (Figure 3).

**Figure 2:**
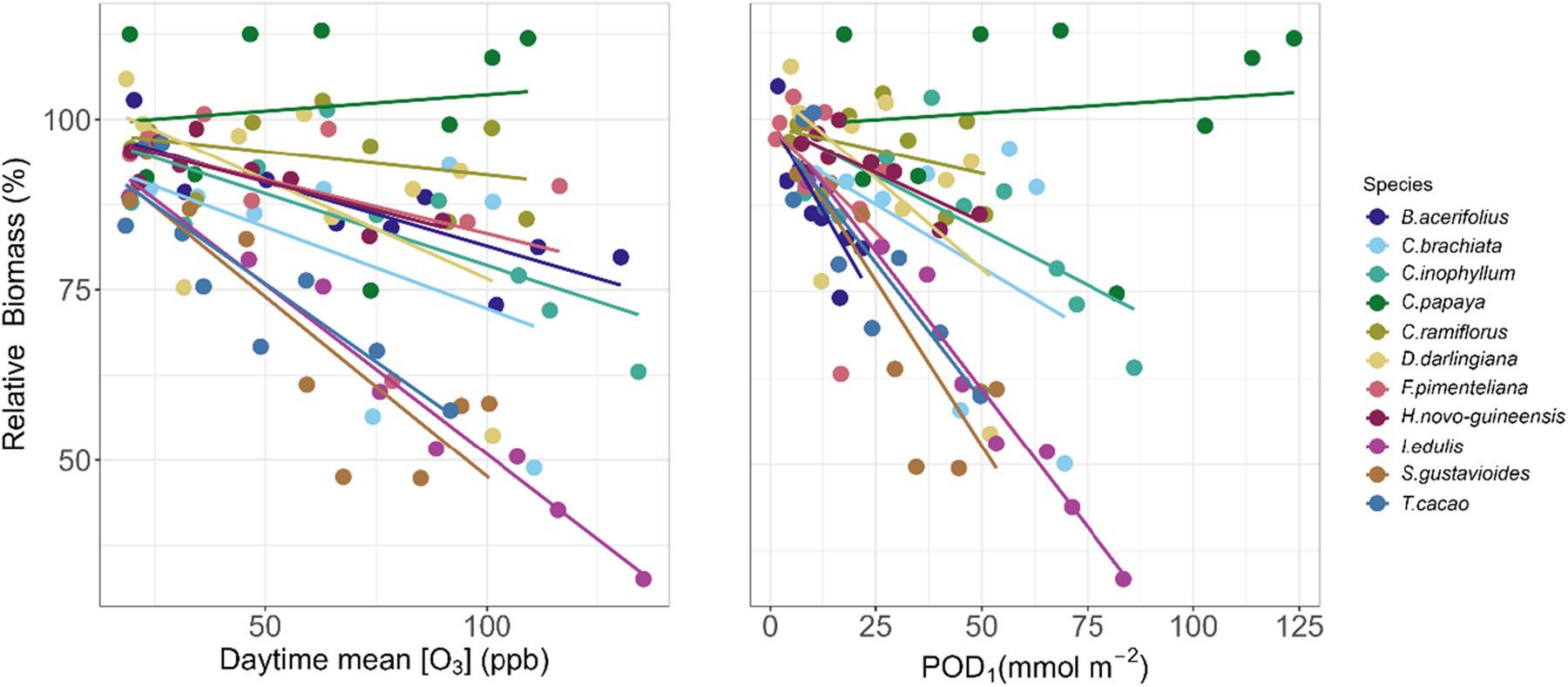
Change in relative plant biomass of 11 tropical tree species with increasing O_3_ exposure. Values represent chamber averages as compared to daytime mean O_3_ concentrations ([O_3_]_day_) and flux‐based metric POD_1_ (Phytotoxic O_3_ Dose ‐ the accumulated stomatal O_3_ flux above a threshold of 1 nmol O_3_ m^−2^ projected leaf area (PLA) s^−1^). [Color figure can be viewed at wileyonlinelibrary.com]

**Figure 3:**
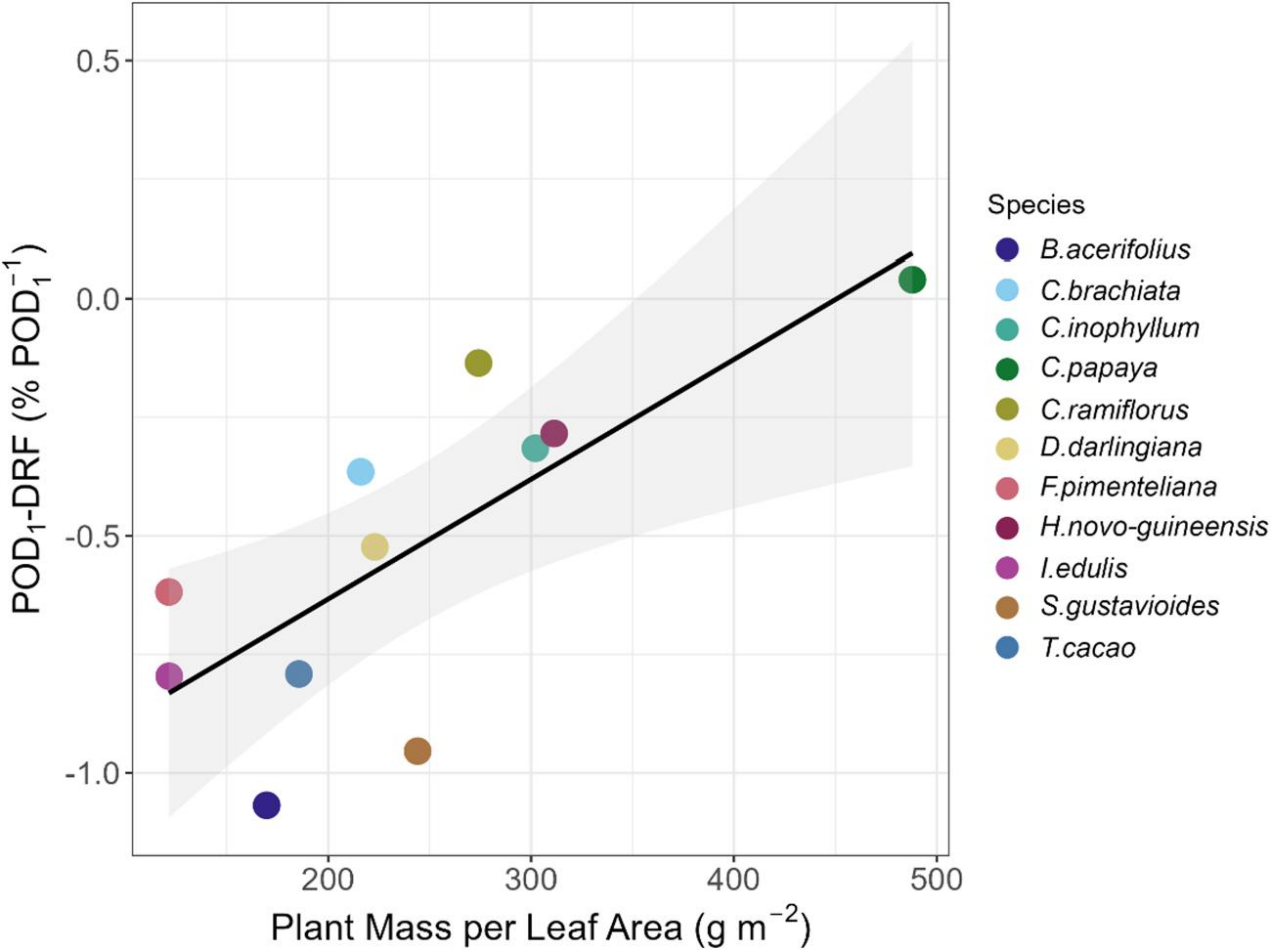
Relationship between the rate of change in biomass with increasing phytotoxic O_3_ dose (POD_1_ Dose Response Function, % POD_1_^−1^) and plant mass per unit leaf area (PMA) across 11 tropical tree species. Regression line and grey ribbon indicate fitted linear model predictions and 95% confidence intervals, respectively, derived from models fitted to unstandardised values; standardised coefficients are reported in the text. [Color figure can be viewed at wileyonlinelibrary.com]

## 4 Discussion

Tropical forests are critical to the global carbon cycle, yet their response to rising tropospheric O_3_ remains poorly understood. Recent work (Cheesman et al. 2024) has highlighted the potential for O_3_ to suppress tropical forest productivity at a global scale, but the mechanisms driving inter‐specific variation in O_3_ responses remain unresolved. In this study, we sought to link variation in leaf traits and biomass allocation to species‐level responses during O_3_ exposure, providing new insight into the trait‐based determinants of O_3_ susceptibility in tropical trees. We assessed the O_3_ susceptibility of 11 tropical tree species grown in open top chambers under a range of [O_3_] and measured plant biomass responses with main plant functional traits such as plant mass per area (PMA), leaf mass per area (LMA), total phenolic content (TPC), and intrinsic efficiency (iWUE). We found O_3_ exposure had a significant negative impact on biomass accumulation, and root‐to‐shoot ratio (R:S). This indicates that these tropical tree species are susceptible to O_3_, and that there are species‐level differences in O_3_ susceptibility. We also found that the structural trait PMA was a significant factor in explaining the O_3_ susceptibility among the tropical tree species.

### 4.1 Changing Leaf Traits With Elevated O_3_ Exposure

Our study found that both TAC and TPC varied substantially among the species tested, and that they were significantly corelated across all sampled leaves—consistent with the role of phenolics as key contributors to antioxidant defence in plant tissues (Grace 2005; Dudonne et al. 2009). However, we found that neither TAC nor TPC showed a consistent relationship with increasing O_3_ exposure, across species. These findings contrast with several studies in temperate trees that have observed increases in response to O_3_ exposure (Yamaji et al. 2003; Gao et al. 2016). Yet our results are perhaps more consistent with the limited antioxidant response observed in the tropical tree *Eugenia uniflora* when exposed to free‐air O_3_ (da Silva Engela et al. 2021). A possible explanation for this pattern is that TPC represents a composite measure of a diverse group of compounds that vary widely in their antioxidant capacity (Grace 2005). Thus, changes in TPC may not reflect effective antioxidant defence under O_3_ stress. Instead, individual phenolic compounds can respond variably to O_3_ exposure, with some increasing while others decline. In birch, O_3_ exposure has been shown to induce increases in specific phenolic compounds including phenolic acids and flavonoids (Yamaji et al. 2003; Kontunen‐Soppela et al. 2007). It is also important to note that the complex interactions between observed oxidative response and leaf‐age (Farha et al. 2023)—even within cultivars of the same species (Pisuttu et al. 2024)—may limit the utility of intraspecific comparisons made on a single‐age cohort of leaves taken per species. Moreover, the total measured pools of TPC or TAC likely reflect only a portion of the antioxidant function, as dynamic processes such as recycling of antioxidants, subcellular compartmentalisation, and interactions with other antioxidant systems can play crucial roles in mitigating oxidative stress (Dusart et al. 2019).

Across the 11 species examined here, we captured a large range in LMA_Lamina_ (40–143 g m^−2^), yet we observed no statistically significant relationship between changes in the leaf LMA_Lamina_ and O_3_ exposure. This is consistent with previous studies in Banana (Musa cv. Williams, (Farha et al. 2023)), several C_4_ grasses (Li et al. 2022; Farha et al. 2025), and a broad meta‐analysis of LMA conducted by Poorter et al. (2009). Nevertheless, other studies have reported significant negative effects of O_3_ on LMA in both poplar (*Populus* spp.) clones (Shang et al. 2017) and sugarcane genotypes (Cheesman et al. 2023)—suggesting that the impact of O_3_ on LMA may be species dependant.

Intrinsic water‐use efficiency (iWUE), the ratio of photosynthetic rate to *g*_s._, reflects the balance between carbon gain and water loss regulation at the leaf level, as such iWUE serves as a key indicator of stomatal behaviour in response to changing environmental conditions. Many studies have reported a decline in iWUE under O_3_ exposure (Hoshika et al. 2015; Xu et al. 2020; Li et al. 2021; Xu et al. 2021) due to O_3_‐induced reductions in photosynthesis, impaired stomatal regulation, or changes in the coupling of photosynthesis and *g*_s_ (Cernusak et al. 2021). However, other studies agree with our findings and have found no effect of O_3_ on iWUE (Zhang et al. 2014; Sugai et al. 2018), which may reflect the well‐watered nature of our experimental trees.

### 4.2 Changes in Plant Architecture Due to O_3_ Exposure

Exposure to O_3_ significantly altered biomass allocation in the tropical saplings examined. Both increasing [O_3_]_day_ and POD_1_ were associated with an increase in LMR and a concurrent decrease in R:S, indicating a shift in carbon allocation towards aboveground tissues. This pattern suggests that plants may prioritise leaf investment under O_3_ stress, potentially to maintain photosynthetic capacity when exposed to oxidative stress. In *Betula* and *Populus*, O_3_‐induced growth reductions were associated with lower R:S, reflecting reduced allocation to belowground biomass (Oksanen et al. 2009). Similar shifts in biomass allocation have been reported in temperate angiosperms (Landolt et al. 2000), supporting the idea that O_3_ exposure can drive changes in tree architecture, with potential implications for water use and nutrient acquisition. However, such changes must be interpreted cautiously in immature trees as they may also reflect ontogenetic effects rather than purely stress‐driven responses. In young saplings, changes in LMR and R:S can occur naturally as plants progress from establishment to rapid aboveground growth, independent of environmental stressors (Poorter et al. 2012). Given that we also observed differences in total biomass (see below), the shift in allocation may represent a typical rebalancing of growth priorities rather than a direct stress response. Further work examining differences in growth and biomass allocation over time under different levels of O_3_ exposure would be required to disentangle these potential mechanisms.

### 4.3 Tropical Tree Susceptibility to O_3_

Examining the change in total biomass at the end of the experimental growth‐period showed that O_3_ exposure at biologically relevant levels generally had a negative impact on the biomass of tropical trees—and that there were significant species‐level differences in O_3_ susceptibility when considering both [O_3_]_day_ and POD_1_ as metrics of O_3_ exposure. Two species (*C. papaya* and *C. ramiflorus*) were found to be apparently O_3_ tolerant with no significant change in final biomass. However, on average across the 11 tropical trees examined the average POD_1_ dose response function of 0.52% POD_1_^−1^ (IQR − 0.79 to −0.30% POD_1_^−1^); this is comparable to the range seen in highly studied temperate and mediterranean tree species (Hu et al. 2015; CLRTAP et al. 2017). It is important to note here that this average dose response is the same as that of the ‘moderate’ O_3_ susceptibility (i.e. 0.50% POD_1_) used to model tropical forests in Cheesman et al. (2024)—which although using much of the same underlying data—utilised a different coupled photosynthesis‐stomatal model to predict *g*_s_ and calculate POD_1_ during experimental exposure.

### 4.4 Interspecific Variation in O_3_ Susceptibility

Studies have consistently observed substantial inter‐specific (CLRTAP et al. 2017; Marzuoli et al. 2018) and even cultivar‐level (Shang et al. 2017) differences in O_3_ susceptibility of tree species. Given the need to extrapolate an understanding of O_3_ susceptibility to untested species there is an obvious motive to examine it within a functional trait framework (Bussotti 2008; Ma et al. 2023). Indeed a meta‐analysis for subtropical, temperate, and Mediterranean tree species appeared to show that differences in O_3_ susceptibility could be ascribed to simple differences in LMA (Feng et al. 2018). However, subsequent studies have shown that intraspecific variation in O_3_ susceptibility of Poplar cultivars cannot be ascribed to leaf morphological traits such as LMA (Shang et al. 2020), nor does there seem to be a direct effect of LMA on O_3_ susceptibility in a range of generally O_3_ tolerant C_4_ crops (Li et al. 2022). It should also be noted that much of the link between LMA and interspecific variation in O_3_ susceptibility reported by Feng et al.(2018) may also represent a distinction between low‐LMA angiosperms and high‐LMA gymnosperms. Our data examining O_3_ susceptibility in tropical angiosperms does not show evidence for LMA being a strong determinant of species‐specific O_3_ responses. Indeed, across our diverse range of species we found no evidence of any leaf‐level functional traits measured including TPC, iWUE or N‐leaf being related to observed O_3_ susceptibility.

Our analysis identified PMA as the only consistently significant predictor of O_3_ susceptibility, indicating that species with greater total biomass per unit leaf area were less affected by O_3_, whilst those with higher leaf area relative to total biomass were more susceptible. Although this conclusion is based on just 11 diverse tropical tree species, the absence of any clear signal from leaf‐level physiological or biochemical traits, and the emergence instead of a whole‐plant structural characteristic as the primary determinant, suggests that plant responses to oxidative stress are inherently multifaceted, and that perhaps no single leaf‐level trait is able to capture this complexity across the hyper‐diverse tropical flora. It is plausible that with a larger sample of species, additional functional traits may emerge as significant predictors, and the strength and generality of the PMA relationship could be better resolved. However, our current findings have implications for how we think about tropical forest vulnerability to rising tropospheric O_3_: a community comprising of species with a wide diversity of structural and functional strategies to deal with oxidative stress will collectively dampen the ecosystem‐level signal of O_3_‐induced damage relative to what would be predicted from any single representative species. Future efforts to model the impacts of O_3_ on tropical forests should therefore seek to represent this diversity in mixed‐species dynamic forest models able to better simultaneously integrate a diversity of responses to a range of environmental drivers (Maréchaux et al. 2025).

## Conflicts of Interest

The authors declare no conflicts of interest.

## Supporting information


Supporting File

## Data Availability

The data that support the findings of this study are openly available in Dryad data repository at https://doi.org/10.5061/dryad.4b8gthtmz.
