# Supplementary material for: Ozone Susceptibility of Tropical Tree Species
Source: Plant Cell Environ. 2026 Aug 2;49(11):8883–94. doi: 10.1111/pce.70777 (PMC13646691; doi:10.1111/pce.70777)
Supplement: Supplementary file 1 [file PCE-49-8883-s001.docx]

**Article title:** Ozone Susceptibility of Tropical Tree Species

**Authors:** Mst Nahid Farha, Flossie Brown, Lucas A. Cernusak, Stephen Sitch, Alexander W. Cheesman

The following Supporting Information is available for this article:

**Table S1** Species-specific stomatal conductance parameterization applied in DO_3_SE (Deposition of ozone for Stomatal Exchange).

**Table S2** Environmental conditions in Open Top Chambers over the course of ozone exposure experiments.

**Table S3** Daytime mean ozone concentrations, [O_3_] day, POD_1_ and AOT_40_ observed in nine Open Top Chambers over the experimental periods.

**Figure S1** Effects of ozone exposure on leaf mass ratio and root-to-shoot ratio in tropical tree saplings grown under a range of O3 exposures.

**Figure S2** Pairwise relationships among leaf- and plant-level functional traits across eleven tropical tree species

**Table S1** Species stomatal conductance parameterization applied in DO_3_SE (Deposition of ozone for Stomatal Exchange) model to calculate Phytotoxic Ozone Dose (POD). *g*_max_ is the maximum conductance, f_min_ is the fraction of *g*_max_ at minimum stomatal conductance (*g*_min_). L_d_ the effective leaf blade width, f_temp_, f_light_, f_VPD_ are the functions of *g*_s_ response to air temperature (T, °C), photosynthetically active radiation at the leaf surface (PAR, μmol m^−2^ s^−1^), and vapour pressure deficit (VPD, kPa) respectively. Species stomatal conductance parameterization applied in DO_3_SE (Deposition of ozone for Stomatal Exchange) model to calculate Phytotoxic Ozone Dose (POD). *g*_max_ is the maximum conductance, fmin is the fraction of *g*_max_ at minimum stomatal conductance (*g*_min_). L_d_ the effective leaf blade width, f_temp_, f_light_, f_VPD_ are the functions of *g*_s_ response to air temperature (T, °C), photosynthetically active radiation at the leaf surface (PAR, μmol m^−2^ s^−1^), and vapour pressure deficit (VPD, kPa) respectively.

| Species | *g*_max_ | L_d_ | f_min_ | f_PAR_ | f_VPD_ | | f_temp_ | | |
| --- | --- | --- | --- | --- | --- | --- | --- | --- | --- |
|  |  |  |  |  | VPD_min_ | VPD_max_ | T_min_ | T_opt_ | T_max_ |
|  | (mmol O­_3_ m^-2^ s^‑1^) | (m) |  |  | (kPa) | (kPa) | (°C) | (°C) | (°C) |
| *B. acerifolius* | 135 | 0.13 | 0.07 | 0.016 | 4.5 | 1.9 | 12 | 27 | 45 |
| *C. brachiata* | 166 | 0.05 | 0.07 | 0.006 | 5 | 2.5 | 15 | 32 | 49 |
| *C. inophyllum* | 184 | 0.08 | 0.11 | 0.005 | 4 | 2.8 | 14 | 30 | 45 |
| *C. papaya* | 268 | 0.05 | 0.01 | 0.01 | 6 | 3.5 | 15 | 30 | 51 |
| *C. ramiflorus* | 126 | 0.08 | 0.07 | 0.007 | 5 | 2 | 17 | 32 | 47 |
| *D. darlingiana* | 154 | 0.12 | 0.06 | 0.008 | 3.6 | 2.3 | 20 | 32 | 45 |
| *F. pimentelia* | 79 | 0.03 | 0.09 | 0.01 | 5 | 2.5 | 17 | 30 | 45 |
| *H. novo-guineensis* | 296 | 0.32 | 0.03 | 0.004 | 7 | 2.5 | 20 | 33 | 46 |
| *I. edulis* | 279 | 0.08 | 0.19 | 0.01 | 3.8 | 2 | 15 | 30 | 45 |
| *S.gustavioides* | 149 | 0.05 | 0.03 | 0.013 | 3.7 | 2.5 | 14 | 30 | 45 |
| *T. cacao* | 167 | 0.12 | 0.02 | 0.008 | 6 | 2.5 | 20 | 35 | 50 |

**Table S2** Environmental conditions in Open Top Chambers over the course of ozone exposure experiments. Data represents an average (± 1 SD) of daily daytime (08:00 to 17:00 h) average.

| Species | Air temperature (T) | | | Relative humidity (RH) | | | Photosynthetically active radiation (PAR) | | |
| --- | --- | --- | --- | --- | --- | --- | --- | --- | --- |
|  | (°C) | | | (%) | | | (µmol m^-2^ s^-1^) | | |
| *B. acerifolius* | 30.6 | ± | 2.6 | 59.0 | ± | 9.6 | 1356 | ± | 676 |
| *C. brachiata* | 28.2 | ± | 3.6 | 63.4 | ± | 11.2 | 1025 | ± | 603 |
| *C. inophyllum* | 28.0 | ± | 2.5 | 59.5 | ± | 11.6 | 1086 | ± | 604 |
| *C. papaya* | 28.5 | ± | 3.6 | 63.7 | ± | 11.0 | 1031 | ± | 601 |
| *C. ramiflorus* | 28.2 | ± | 3.5 | 63.6 | ± | 11.1 | 1019 | ± | 599 |
| *D. darlingiana* | 27.8 | ± | 3.3 | 65.8 | ± | 12.7 | 938 | ± | 563 |
| *F. pimentelia* | 29.9 | ± | 3.2 | 62.3 | ± | 10.1 | 1161 | ± | 640 |
| *H. novo-guineensis* | 29.6 | ± | 3.4 | 57.4 | ± | 11.9 | 1206 | ± | 674 |
| *I. edulis* | 26.8 | ± | 2.8 | 66.6 | ± | 13.3 | 882 | ± | 508 |
| *S. gustavioides* | 27.3 | ± | 3.1 | 65.0 | ± | 12.1 | 935 | ± | 556 |
| *T. cacao* | 30.1 | ± | 3.5 | 59.3 | ± | 12.7 | 1191 | ± | 675 |

**Table S3** Daytime mean ozone concentrations, [O_3_]_day_, POD_1_ ((Phytotoxic O_3_ Dose - the accumulated stomatal O_3_ flux above a threshold of 1 nmol O_3_ m^−2^ projected leaf area (PLA) s^−1^) and AOT_40_ (accumulated O_3_ concentrations over a threshold of 40 ppb) observed in nine Open Top Chambers over the experimental periods. [O_3_]_day_ (standard deviations are shown in brackets) data from between 08:00 and 17:00 local time over the entire experiments.

| Ozone  metrics | Species | Chamber | | | | | | | | |
| --- | --- | --- | --- | --- | --- | --- | --- | --- | --- | --- |
|  |  | 1 | 2 | 3 | 4 | 5 | 6 | 7 | 8 | 9 |
| [O_3_]_day_ (ppb) | | | | | | | | | | |
|  | *B. acerifolius* | 20.3 (5.3) | 31.7 (7.4) | 50.3 (10.9) | 66.0 (16.8) | 78.4 (18.3) | 86.0 (21.8) | 102.1 (26.0) | 111.7 (32.2) | 130.3 (34.7) |
|  | *C. brachiata* | 19.8 (5.0) | 23.9 (5.6) | 34.7 (10.7) | 47.6 (15.1) | 63.2 (21.9) | 74.2 (28.2) | 91.5 (34.6) | 101.3 (38.4) | 110.8 (43.5) |
|  | *C. inophyllum* | 19.6 (4.8) | 31.8 (6.1) | 48.3 (9.1) | 64.0 (11.2) | 75.1 (14.1) | 89.1 (15.2) | 107.3 (17.9) | 114.3 (26.3) | 134.3 (24.9) |
|  | *C. papaya* | 19.3(5.1) | 23.1(6.0) | 34.1 (10.4) | 46.6 (14.8) | 62.7 (21.6) | 73.8 (27.6) | 91.5 (34.5) | 101.2 (38.5) | 109.4 (44.9) |
|  | *C. ramiflorus* | 19.6 (5.0) | 23.6 (5.7) | 34.4 (10.7) | 47.2 (15.0) | 62.9 (21.6) | 73.7 (27.9) | 91.3 (34.3) | 101.1 (38.0) | 109.1 (44.9) |
|  | *D. darlingiana* | 18.5 (5.5) | 22.4 (6.1) | 31.5 (11.2) | 44.1 (15.4) | 58.8 (20.6) | 65.0 (27.2) | 83.3 (32.6) | 93.9 (35.9) | 101.2 (41.9) |
|  | *F. pimentelia* | 19.4 (5.1) | 23.3 (6.4) | 36.2 (10.8) | 47.0 (16.1) | 64.3 (24.0) | 78.7 (29.2) | 95.6 (37.7) | 104.1 (43.3) | 116.5 (47.6) |
|  | *H. novo-guineensis* | 19.6 (4.9) | 21.3 (6.0) | 30.6 (10.1) | 26.0 (7.8) | 34.4 (11.6) | 46.9 (16.6) | 55.8 (23.4) | 73.5 (31.2) | 90.0 (40.2) |
|  | *I. edulis* | 19.2 (4.3) | 31.6 (5.2) | 46.3 (7.7) | 63.1 (10.5) | 75.9 (13.2) | 88.5 (14.8) | 107.0 (16.8) | 116.1 (22.7) | 135.4 (23.4) |
|  | *S. gustavioides* | 19.3 (5.4) | 23.2 (6.1) | 32.9 (11.6) | 45.8 (16.2) | 59.4 (21.9) | 67.6 (28.5) | 85.0 (34.5) | 94.2 (38.2) | 100.5 (44.3) |
|  | *T. cacao* | 18.4 (5.2) | 22.0 (7.0) | 31.2 (10.9) | 26.5 (11.4) | 36.1 (13.3) | 49.0 (17.7) | 59.2 (25.6) | 75.2 (30.8) | 91.7 (38.8) |
| AOT_40_ (ppm-h) | | | | | | | | | | |
|  | *B. acerifolius* | 0.0 | 0.1 | 6.4 | 15.2 | 26.0 | 21.8 | 34.8 | 40.1 | 50.2 |
|  | *C. brachiata* | 0.0 | 0.0 | 3.4 | 16.6 | 57.9 | 40.6 | 85.1 | 100.5 | 115.2 |
|  | *C. inophyllum* | 0.0 | 0.4 | 13.8 | 36.6 | 74.7 | 53.8 | 102.2 | 113.6 | 143.2 |
|  | *C. papaya* | 0.0 | 0.0 | 3.4 | 17.2 | 44.7 | 64.0 | 95.4 | 112.6 | 127.5 |
|  | *C. ramiflorus* | 0.0 | 0.0 | 3.4 | 16.7 | 59.1 | 41.6 | 87.8 | 103.8 | 117.4 |
|  | *D. darlingiana* | 0.0 | 0.0 | 3.3 | 13.6 | 47.9 | 36.4 | 77.9 | 96.1 | 109.2 |
|  | *F. pimentelia* | 0.0 | 0.0 | 2.7 | 11.9 | 46.0 | 30.5 | 64.7 | 74.5 | 87.8 |
|  | *H. novo-guineensis* | 0.0 | 0.1 | 0.9 | 0.1 | 14.5 | 2.7 | 26.1 | 48.8 | 70.3 |
|  | *I. edulis* | 0.0 | 0.1 | 7.3 | 23.8 | 49.7 | 36.8 | 68.4 | 78.0 | 97.4 |
|  | *S. gustavioides* | 0.0 | 0.0 | 3.2 | 13.4 | 43.2 | 31.3 | 67.1 | 80.0 | 89.4 |
|  | *T. cacao* | 0.0 | 0.1 | 1.7 | 1.8 | 20.7 | 5.7 | 37.6 | 62.9 | 89.7 |
| POD_1_ (mmol m^-2^) | | | | | | | | | | |
|  | *B. acerifolius* | 1.8 | 4.0 | 7.2 | 10.0 | 12.2 | 13.7 | 16.6 | 18.1 | 21.7 |
|  | *C. brachiata* | 8.0 | 10.7 | 18.1 | 26.6 | 37.0 | 45.0 | 56.5 | 62.9 | 69.5 |
|  | *C. inophyllum* | 8.2 | 16.3 | 27.5 | 38.2 | 45.8 | 55.2 | 67.7 | 72.4 | 86.1 |
|  | *C. papaya* | 17.6 | 22.0 | 35.0 | 49.7 | 68.5 | 82.0 | 102.8 | 113.9 | 123.7 |
|  | *C. ramiflorus* | 4.6 | 6.6 | 12.1 | 18.7 | 26.7 | 32.6 | 41.6 | 46.5 | 50.8 |
|  | *D. darlingiana* | 5.0 | 7.1 | 12.2 | 19.3 | 27.4 | 31.3 | 41.6 | 47.5 | 52.0 |
|  | *F. pimentelia* | 1.3 | 2.3 | 5.6 | 8.5 | 13.0 | 16.8 | 21.2 | 23.5 | 26.8 |
|  | *H. novo-guineensis* | 7.5 | 8.6 | 13.9 | 11.2 | 16.4 | 23.9 | 29.3 | 40.1 | 49.5 |
|  | *I. edulis* | 9.0 | 16.9 | 26.4 | 37.1 | 45.4 | 53.4 | 65.3 | 71.3 | 83.5 |
|  | *S. gustavioides* | 6.5 | 8.7 | 14.3 | 21.8 | 29.5 | 34.6 | 44.6 | 49.7 | 53.4 |
|  | *T. cacao* | 5.6 | 8.0 | 13.0 | 10.3 | 16.3 | 24.1 | 30.5 | 40.2 | 49.7 |

**Figure S1** Effects of ozone exposure on leaf mass ratio (LMR, a, b) and root-to-shoot ratio (R:S, c, d) in tropical tree saplings grown under a range of O_3_ exposures. Plots contains underlying data and predicted relationships based on two ozone exposure metrics including daytime mean O_3_ (ppb) (a,c) and POD_1_ (Phytotoxic O_3_ Dose - the accumulated stomatal O_3_ flux above a threshold of 1 nmol O_3_ m^−2^ projected leaf area (PLA) s^−1^ (b, d). Dashed lines and grey ribbons indicate predicted means and 95% confidence intervals from linear mixed-effects models fitted with species as a random intercept.

**Figure S2** Pairwise relationships among leaf- and plant-level functional traits across eleven tropical tree species. Panels show scatterplots (lower triangle), smoothed trait distributions (diagonal), and Pearson correlation coefficients (upper triangle) among six traits: leaf mass ratio (LMR), plant mass per unit area (PMA), lamina leaf mass per area (LMA_Lamina_), total phenolic content (TPC), intrinsic water-use efficiency (iWUE), and leaf nitrogen content (N-leaf). Asterisks indicate statistically significant correlations (P < 0.05, P < 0.01). Trait values are species means.
